# Microfluidic colloid filtration

**DOI:** 10.1038/srep22376

**Published:** 2016-03-01

**Authors:** John Linkhorst, Torsten Beckmann, Dennis Go, Alexander J. C. Kuehne, Matthias Wessling

**Affiliations:** 1 RWTH Aachen University, AVT.CVT-Chemical Process Engineering, Aachen, 52056, Germany; 2 DWI–Leibniz Institute for Interactive Materials, Aachen, 52056, Germany

## Abstract

Filtration of natural and colloidal matter is an essential process in today’s water treatment processes. The colloidal matter is retained with the help of micro- and nanoporous synthetic membranes. Colloids are retained in a “cake layer” – often coined fouling layer. Membrane fouling is the most substantial problem in membrane filtration: colloidal and natural matter build-up leads to an increasing resistance and thus decreasing water transport rate through the membrane. Theoretical models exist to describe macroscopically the hydrodynamic resistance of such transport and rejection phenomena; however, visualization of the various phenomena occurring during colloid retention is extremely demanding. Here we present a microfluidics based methodology to follow filter cake build up as well as transport phenomena occuring inside of the fouling layer. The microfluidic colloidal filtration methodology enables the study of complex colloidal jamming, crystallization and melting processes as well as translocation at the single particle level.

Membrane filtration is the essential process in water purification, sterile filtration and bioprocessing. The greatest challenge in membrane filtration is to gain control over the separation process. While the rejection of matter is the essential function of a membrane, the rejected matter poses an continuously increasing resistance in front of the membrane or inside of the membrane. This phenomenon is known as membrane fouling^1^. For the mitigation of fouling effects, membrane research of today focuses almost exclusively on the design of new materials by using fouling resistant materials. However, in a normal filtration process, the interaction between particles and the membrane occurs over a very short initial time. This initial interaction may preordain the entire downstream deposition process. Very little is known about the integral phenomena occurring during layer deposition and its transport processes. Slow aggregation processes can occur as well as fast jamming processes^2^. To gain an understanding of these processes, simulations have been carried out to follow slow aggregation^3^,^4^. Experimental studies have been performed using colloidal particles to study fast jamming in microfluidic channels^5^.

Post filtration analysis of filter cakes is possible, however the real morphology of soft matter based deposits might be obscured as they collapse during drying and at high vacuum conditions. *In-situ* imaging of filter cake formation and their behavior under filtration conditions are hence higly desired. To gain insight into the various fouling processes a variety of optical and non-optical *in situ* analyses have been developed^6^,^7^,^14^. For example, laser sheet microscopy has been utilized to study filter cake formation and to quantify the filter cake height inside of hollow fibre membranes during inside-out filtration^8^. Alternatively, nuclear magnetic resonance (NMR) can also be applied to study fouling^9^. However, these methods facilitate only monitoring of the macroscopic growth of the cake layer and the membrane fouling process and not the visualization of the intricate phenomena occurring at the microscale. By contrast, Confocal Laser Scanning Microscopy (CLSM), can be applied to visualize membrane-particle interactions. CLSM allows discrimination and real-time visualization of different species adsorbing at and desorbing from the membrane surface, enabling *in situ* investigation of fouling at flatsheet membranes^10^. For better understanding of the preponderant processes during fouling, studies at the single pore level have been carried out by clogging of pores produced in a microfludic channel^5^. Microfluidics offers the advantage of well defined and controlled custom geometries generated via lithography in a transparent elastomer. This setup allows for optical observation of fouling and clogging processes *in situ*. A first study found the microfluidic model pores to clog independently of particle flow rate and volume fraction^5^. By contrast, a more recent study describes a critical flux, at which particles form arches in the pores as well as the dependency of salt concentration deposition geometries^11^. This contradiction with the previous observation has not been resolved to date. Real membrane materials can also be studied on microfluidic chips by utilizing phase separation micromolding to prepare a porous membrane-like microchannel geometry^12^. When applying bidisperse colloidal dispersions to these microfluidic chips, an amorphous and homogeneous cake layer forms on the feed side of the membrane^13^.

In essence, overall cake layer formation can be studied on macroscopic membranes; however, the individual particle-pore interaction has not been resolved. By contrast, single particle interactions with defined pores can be studied in microfluidics; however, these systems do not represent real membrane or cake layer geometries sufficiently. The connection between these two regimes is missing, obviating true physical insight into membrane fouling processes. Understanding these processes would enable the design of novel membrane architectures of improved performance with impact in many applications from water purification to fluid stream filtration and energy generation.

Here, we aim at resolving this discrepancy by developing a model system composed of a microfluidic chip featuring a set of parallel microchannels with constrictions to mimic membrane pores.

A model filter cake is built-up using monodisperse soft microgels individually labeled with fluorescent cores forming a densely packed colloidal aggregate under the drag force of the fluid^15^,^16^. The soft microgels represent a realistic cake behaviour especially with regards to biofouling, where a compressible cake layer is produced. We image membrane fouling with the microgels in multiple dimensions and real-time using CLSM. To further showcase the potential of this technique we can track small, hard and fluorescent particles as they enter the filter cake and translocate towards lower pressures following a tortuous path. In combination these techniques facilitate the investigation of various fundamental processes, which we elaborate here one by one.

## Results and Discussion

### Channel clogging

**Figure 1:**
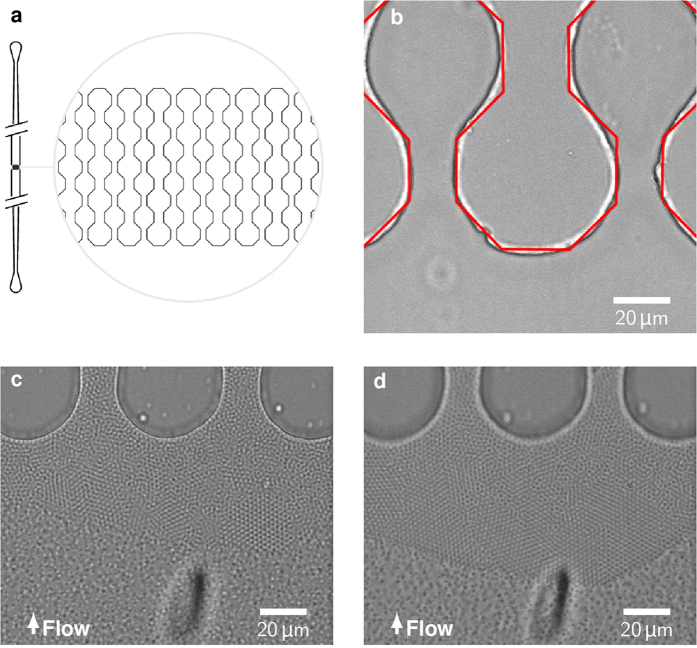
(**a**) Design of the microfluidic chip, according to^5^ (**b**) Dry chip before filtration with red lines marking the original design (**c**) Build-up of the filter cake during filtration of microgel suspension (**d**) Increasing crystallinity under growing pressure drop.

We infuse fluorescently labeled microgels with a diameter of 2.2 μm under constant flux through a 500 μm wide inlet into a parallel set of microchannels with bottleneck constrictions along their length (see Fig. 1a,b). The width of the constrictions and channel height is varied between 8, 11 and 20 μm. The channels are produced in poly-(dimethylsiloxane) (PDMS) using soft lithography and are bonded to a microscope cover glass using plasma treatment. This relatively flat geometry facilitates tracking of particles as they cannot move out of the 3D field of view of a CLSM. The infusion of monodisperse microgels produces a filter cake, which builds up in front of the microchannel constrictions. The microgels partly self-assemble into colloidal crystals with hexagonal lattice as can be seen in Fig. 1c. As expected, the filter cake grows with longer constant flux filtration times and the trans-filter-cake pressure drop increases. The microgels evade stress by rearranging into energetically favorable structures such as larger crystalline regions (Fig. 1d). Exemplary data of pressure evolving during filtration in the chip can be found as Supplementary Figure 1.

### Compaction

Increasing pressure does not only effect the crystallinity, but also the degree of compaction in the filter cake, when it consists of soft colloids. When, using hard particles as a model system for a filter cake the pressure loss is only affected by the height of the filter cake. Our soft microgels present a better model for real-world filter cakes where a mixture of hard and soft material is retained. Much like real-life filter cakes, soft particles can be compressed. Thus, the pressure drop across the filter cake is influenced by the cake size and the compaction state. The reason for an increasing pressure drop with increasing compaction is the shrinkage of the interstitial space between the particles. By using packed beds of monodisperse soft particles as a model filter cake, the pore size distribution during compaction can be determined.

**Figure 2:**
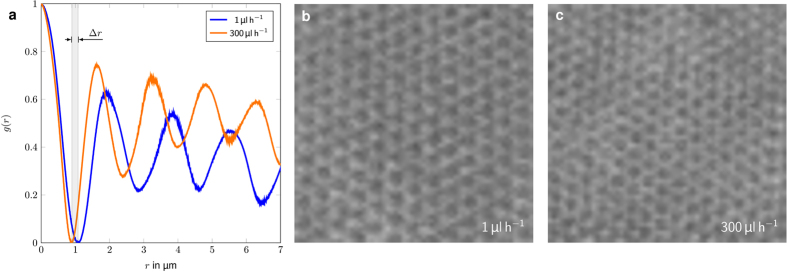
Radial distribution plot of a filter cake at different stages of compaction. The Δ*r* denotes the compression of the center particle. It shrinks from an initial diameter of 2.2 μm to 1.8 μm.

Radial distribution function for crystalline filter cakes at two different compaction stages, resulting from flow rates of 1 μL/h and 300 μL/h are shown in Fig. 2a. While the microgel diameter decreases by 20%, when increasing the flow rate by a factor of 300, the overall crystal structure of the cake remains the same (see Fig. 2b,c). Due to the microgel’s soft nature without a precise interface it is problematic to determine a realistic interstitial void volume. For simplification we assume a spherical shape for the microgels and a uniform deformation of the particles. At 1 µL/h and close to no compression, the calculated interstitial pore size is 340 nm. At a flow rate of 300 µL/h the particle diameter reduces by 20 % to 1.8 µm thus the calculated pore size reduces to 278 nm.

In reality, filter cakes do usually not consist of compacted soft colloidal crystals. In real world membrane fouling the filter cake is often an amorphous deposit due to polydispersity in the colloid size distribution.

### Flow reversal and crystal melting

**Figure 3:**
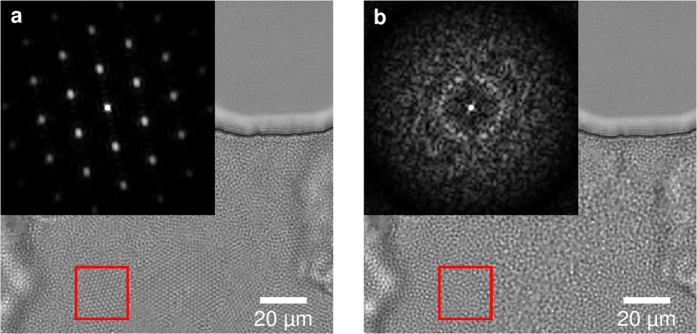
(**a**) Crystalline filter cake formed during first infusion of the microgel suspension. (**b**) Restructured, mostly amorphous filter cake after a short backflush pulse. The insets display the Fourier transform of the red square in the respective image.

To obtain an amorphous cake layer, the soft colloidal crystal is disintegrated by shear induced melting. This is achieved by modulating the flow direction using a short flow reversal, a so called backflush. During backflush the crystallites disintegrate into their original amorphous morphology. Upon switching to a fast forward flow, the microgels jam to form an amorphous cake layer, as shown in Fig. 3. We are also able to form crystallites when using a low forward flow rate instead.

We can monitor the crystallinity by performing Fourier transforms of the images of the crystallized microgels and the backflushed amorphous sample, as shown in the insets of Fig. 3a,b. At first the packed bed of microgels appears ordered in a hexagonal crystal lattice, which is supported by the Fourier transform (Fig. 3a). The crystal lattice is completely molten and the Fourier transform shows the amorphous state in the inset of Fig. 3b.

### Dense colloidal suspension under cross flow

**Figure 4:**
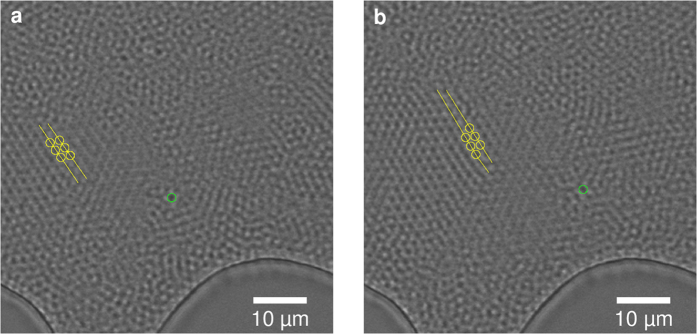
Movement of a crystallite in a dense suspension. The crystallite compensates for cross flow induced shear stress (left to right) by dislocation along the a_1_/a_2_ crystal plane by one particle diameter highlighted in yellow. Suspension flow is visualized by a reference particle in the amorphous region marked with a green circle.

When performing a slow backflush, intact crystallites can be detached from the filter cake. Their behaviour in a dense colloidal suspension can be visualized under filtration conditions. After introducing cross flow the behaviour of such crystallites under shear can be observed. The amorphous particles can easily adjust to the shear force by different speeds of single particles. However, the crystallites deform as a response to the induced shear. In an isolated case we could observe how the crystallite adjusts to the shear force by dislocating along a crystal plane, as indicated in Fig. 4. The sheared-off lattice axis is marked in yellow. The green circle marks a reference particle transported in the amorphous region.

While these effects all occur for particle ensembles and therefore on the macroscale, our methodology also allows for monitoring of single particle effects.

### Particle translocation

**Figure 5:**
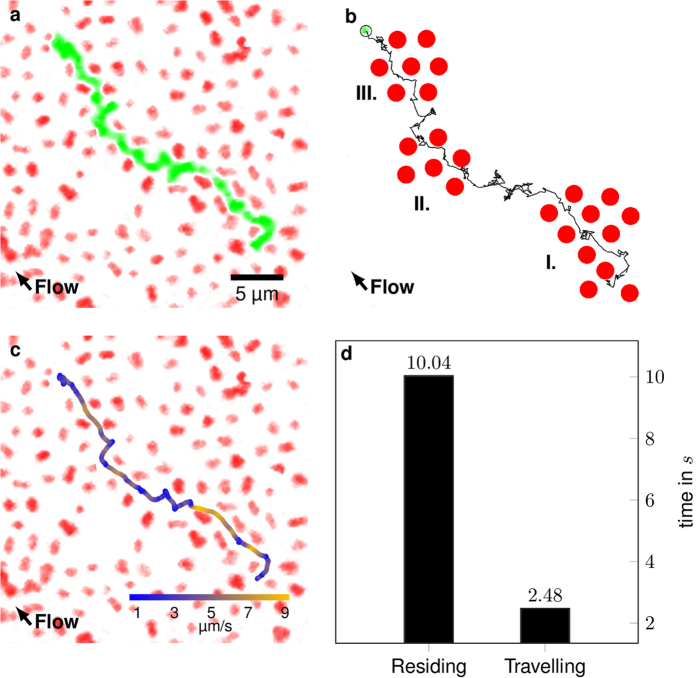
(**a**) Tortuous path of a small, fluorescent tracer particle. The image is stack from 600 individual microscope images. The total travel time for the observed particle is 12.52 s. (**b**) Tracked path of the fluorescent tracer particle with regions of short range order I, II and III. (**c**) Speed color coded path through the partly crystalline bed. (**d**) Average time residing in voids and travelling. Stationary particles and a second tracked particle were masked for clarity. The original video can be found as Supplementary Video 1.

The developed system presents a model for soft cake layers, enabling observation and investigation of transport phenomena inside of deposits on membranes resulting from fouling or filtration effects. We apply small fluorescent tracer particles with a diameter of 300 nm and study their translocation through the mostly amorphous microgel assembly. The small particles fluoresce at a different wavelength than the stationary microgel assembly, allowing us to image the two types of particles at an individual particle level using separate detection channels. While infusing the small particles into the microfluidic system and the jammed microgel assembly, they translocate through the mostly amorphous microgel assembly by following a tortuous path, as shown in Fig. 5a. Comparing the fluorescence data with the respective brightfield image reveals voids in the microgel assembly. These voids are in fact not empty space but microgel particles which have bleached and lost their fluorescence. We here chose to neglect any tracer particles which interact with non-fluorescent microgels and only look at particles, which translocate through the voids generated by fluorescent microgels.

Furthermore we observe that the tracer particles preferentially translocate through microgel assemblies, which exhibit at least short range order, as shown in Fig. 5b with areas of short range order I, II and III. This could be due to the fact that in crystalline areas the voids are aligned as channels, making it easier for the tracer particles to translocate in the direction of the crystallite planes. Alternatively, the microgel motion (similar to phonons in a crystal) could be concerted, making it easier for the tracer particles to slip through the voids, when the microgels move synchroneously. Our method also allows the investigation of velocities of tracer paticles inside of a model filter cake. From the confocal microscopy time-series we can color code the velocity of the tracer particles along their tortuous path, see Fig. 5c. Our previous observation of particles travelling preferentially along more ordered void channels is substantiated by this method. The particle moves faster in domains with short range order and is slowed down in amorphous region. The particle moves fast (orange) where the interstitial channels are straight and roughly aligned with the flow direction. The particle stops at alignment defects of the filter cake until following another interstitial channel in the direction of the flow. The ratio of time residing in voids and time travelling in interstitial channels is 4:1 (see Fig. 5d). The smallest interstitial pores are found in crystals, whereas the amorphous region has larger pores due to non-optimal alignment of the particles. This data leads us to the conclusion that particle translocation through a crystal is favoured over transport through amorphous regions, even though the pores are smaller than in the amorphous regions.

Yet another reason may be that the ordered microgels are more relaxed comparable to the equilibrium state of the microgel in a dilute solution. In contrast, microgels in an non-equilibrium disordered state may be deformed and hence densified: they present an impermeable barrier that can only be passed when (slow) relaxations of the microgel occur, or by meandering around the obstacle.

Depending on the type of membrane and the type of filtered matter, translocation through a filter cake will be slow if it consists of irregularly packed matter with higher free volume and bigger voids. This is counter intuitive as one would expect an irregular filter cake to transport matter faster as its packing density is lower, resulting in a higher volume flow rate. However, particle transport seems to benefit from the aligned smaller pores in a regular crystalline structure. Additionally, there are two opposing hydrodynamic effects. The ordered structure of the microgel crystallites lowers hydraulic resistance compared to an amorphous microgel matrix due to lower tortuosity. By contrast, the packing density of the amorphous matrix is lower than in the crystallites, thus lowering the hydraulic resistance due to less fluid-particle interaction. Which of the two effects is dominant remains to be elucidated, especially because the interaction between water and the microgel is different from thoroughly investigated interactions of classical column packings and water. Further research will have to be conducted to quantify these effects and demonstrate the effect of particle and void sizes as well as their ratio. It is assumed that there is a critical ratio, at which a transition between crystal-preferred and amorphous-preferred transport occurs.

**Figure 6:**
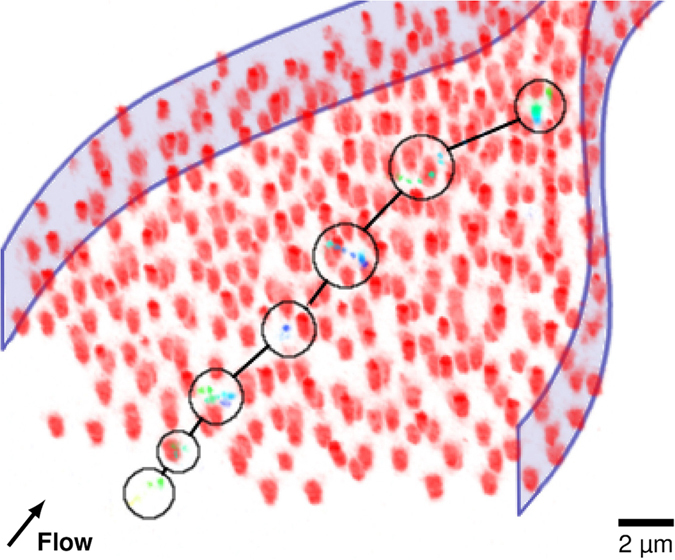
3D particle tracking in a microchannel. The red particles form a filter cake. The circles and lines mark the positions and jumps of the tracked particle, respectively. The color of the tracer particle depicts its z-height in the channel (from bottom/low: dark blue to top/high: green). Stationary particles and a second tracked particle are masked for clarity. The channel walls are indicated as a guide to the eye. The original video can be found as Supplementary Video 2.

While we tracked the previous particle only in 2-D, most of the particles will actually escape the focus plane in the z-direction. At low flow rates we can also track tracer particles in our 3-dimensional microgel filter cake model. Due to fast motion of the tracer beads and scanning rate limitations of the confocal microscope only a limited number of positions can be tracked, as shown in Fig. 6. Nonetheless a tortuous path through the cake layer can be generated from the available data. We color code the z-location of the particles to follow their position in x,y as well as z-directions. In amorphous areas where the particle gets trapped and “resides” for a limited amount of time the tracer particle bounces around, changing also in the z-direction, see Fig. 6.

## Conclusion

The method presented here represents an ideal and unprecedented model, much closer to real world filter cakes than previously reported models and studies. The model consists much like real filter cake of soft and deformable matter, with mixed amorphous and crystalline domains. Our microgels represent idealized foulants, as they can be characterized and optically traced (with their fluorescent core) much like hard particles and they are soft like complex real world organic foulants. Additionally their softness can be tuned by the degree of crosslinking and their surface chemistry can be modified. The filter cake can be modified to match a real world filtration case by setting a certain crystallinity through backflushes and compaction. The system enables investigation of the translocation and interaction of small matter with the soft filter cake, both at the macroscale and the single particle level. We are able to measure position and speed of small particles during transport through the cake layer of soft microgels. These observations made possible through the method presented are relevant for understanding filter cake filtration properties. In the future, this method will enable us to extract permeation performance in relation to the filter cake morphology in a membrane filtration.

While our research is intended to help understanding membrane filtration, the method offers the opportunity to study a variety of other problems. For instance, virus trafficking through vascular tissue remains an unsolved issue in cell biology^17^. Using microgels of different crosslinking density a model cell array could be formed, while smaller labeled particles could act as a model virus particle moving through the model tissue, where also the question whether size-exclusion or charge-exclusion is the predominant effect has to be answered.

## Experimentals

**Figure 7:**
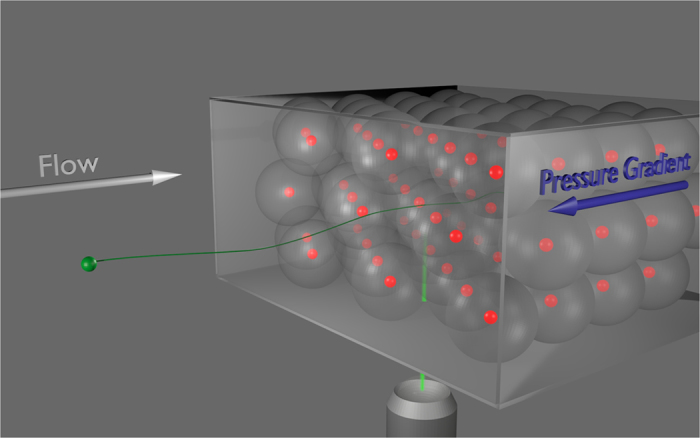
3D scheme of the setup.

We fabricate the microfluidic channel geometries via soft lithography in PDMS. The microfluidic chip is connected to a syringe pump (Chemyx Nexus 3000) using polyethylene tubing. The dispersion of microgels is fed into the chip using the syringe pump. The morphology of the filter cake and the path of the smaller hard particles is observed using a confocal laser scanning microscope (Leica SP8) (see Fig. 7). The channel dimensions are width = 500 μm and height = 11 μm.

The particles used for the filter cake are core-shell soft microgels. The core is made from poly(2,2,2-triuoroethyl mmethacrylate) (PMMA) labeled with Nile Red with a diameter of 300 nm. The soft shell is made from Poly(N-isopropylacrylamide) (pNIPAM) using dispersion polymerization to a diameter of 2.2 μm. The hard particles are essentially cores of the previous particles without shells, made from PMMA labeled with Coumarin 153 and a diameter of 300 nm^18^.

**Figure 8:**
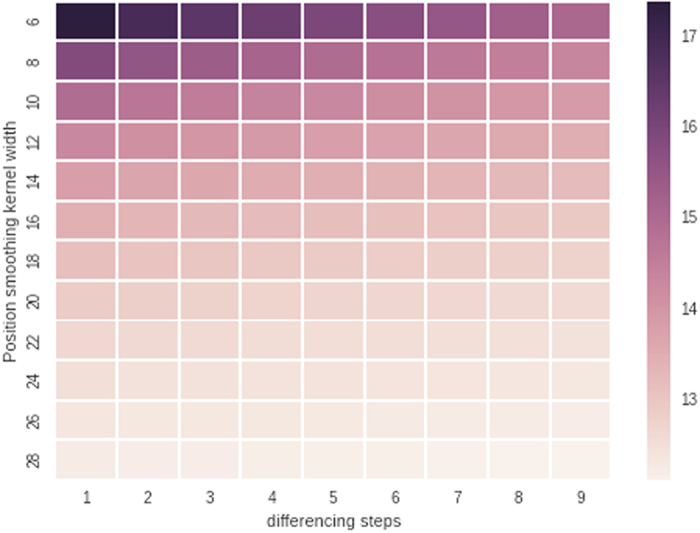
Sensitivity analysis of the tracking and smoothing protocol. Here we chose a kernel width of 10 and a differencing step of 5.

Image analysis is performed using ImageJ and custom python code. ImageJ is used for tracking particle positions, the python libraries pandas and matplotlib are used for post processing of the tracked positions. Smoothing was performed with a filter kernel width of 10 and the particle speed was obtained with a differencing step of 5, using the pandas python library. Sensitivity analysis supports the choice of the kernel and step widths, see Fig. 8.

## Additional Information

**How to cite this article**: Linkhorst, J. *et al.* Microfluidic colloid filtration. *Sci. Rep.* **6**, 22376; doi: 10.1038/srep22376 (2016).
